# Effect of sodium butyrate on kidney and liver mitochondrial dysfunction in a lipopolysaccharide mouse model

**DOI:** 10.1096/fj.202401379RR

**Published:** 2024-12-15

**Authors:** Muznah Khan, Sumera Farooqi, Katherine L. Mitchell, Subir Kumar Roy Chowdhury, Marian Cabrera-Ayala, Jessica Huang, Douglas C. Wallace, Scott L. Weiss

**Affiliations:** 1 Critical Care Mitochondrial Unit, Nemours Biomedical Research, Nemours Children’s Hospital, Wilmington, Delaware, USA; 2 Division of Critical Care, Department of Pediatrics, Nemours Children’s Hospital, Wilmington, Delaware, USA; 3 Center for Mitochondrial and Epigenomic Medicine at the Children’s Hospital of Philadelphia, Philadelphia, Pennsylvania, USA; 4 Division of Human Genetics, Department of Pediatrics, Perelman School of Medicine at the University of Pennsylvania, Philadelphia, Pennsylvania, USA; 5 Department of Pediatrics, Sidney Kimmel Medical College at Thomas Jefferson University, Philadelphia, Pennsylvania, USA; 6 Department of Pathology, Anatomy, and Cell Biology, Sidney Kimmel Medical College at Thomas Jefferson University, Philadelphia, Pennsylvania, USA

**Keywords:** endotoxins, metabolism, mice, microbiota, mitochondria, sepsis

## Abstract

Sodium butyrate can reduce inflammation, but it is not known if butyrate can improve mitochondrial dysfunction during sepsis. We tested butyrate to prevent or reverse lipopolysaccharide (LPS)-induced mitochondrial dysfunction in murine kidney and liver. C57BL/6 mice were grouped as control (*n* = 9), intraperitoneal (IP) LPS (*n* = 8), pretreatment with IP butyrate 600 (*n* = 3) or 1200 mg/kg (*n* = 8) followed 2 h later by LPS, posttreatment with IP butyrate 600 (*n* = 3) or 1200 mg/kg (*n* = 7) 1 h after LPS, or butyrate 1200 mg/kg only (*n* = 8). Kidney and liver tissue were collected at 24 h to measure mitochondrial respiration, electron transport system (ETS) complex activity and subunit expression, and content (citrate synthase [CS] activity and mtDNA/nDNA). Kidney mitochondrial respiration was decreased after LPS compared to controls. Pretreatment with butyrate 1200 mg/kg increased kidney OXPHOS_CI+II_, ETS_CI+II_, ETS_CII_, and CIV respiration compared to LPS; posttreatment did not achieve significant increases except for OXPHOS_CI_. Liver mitochondrial respiration exhibited a similar pattern as in kidney, but differences were not significant. ETS complex and CS activity did not differ between groups, but CI and CII subunit expression trended higher with butyrate in kidney. Changes in mtDNA/nDNA followed a similar pattern as respiration in kidney and liver with a decrease after LPS that was not present with butyrate pretreatment. These data show that butyrate can prevent—but not significantly reverse—the LPS-induced decrease in kidney mitochondrial respiration without a clear effect in liver. Mitochondrial protection was not attributable to changes in ETS complex activity but may reflect maintenance of ETS subunit expression.

## 1 ∣ INTRODUCTION

Sepsis is a life-threatening organ dysfunction caused by a dysregulated response to infection.^1,2^ In the United States, more than 1 million patients are hospitalized with sepsis each year, including >75 000 children.^3^ While most patients improve with early recognition, antimicrobial therapy, and goal-directed resuscitation, mortality rates up to 15%–50% are commonly reported.^4–6^ Although the number and severity of affected organ systems are critical risk factors for sepsis-related death, specific therapies to mitigate or reverse organ dysfunction remain elusive.^7^

Mitochondrial dysfunction has been implicated as a key cellular event contributing to organ dysfunction in sepsis.^8–14^ In animal models and humans with sepsis, mitochondrial dysfunction occurs across organ systems,^12,15–21^ and restoration of mitochondrial function is associated with organ recovery and improved survival.^22–25^ As such, strategies to optimize mitochondrial function present an innovative approach to improve outcomes in sepsis.^26^

Sodium butyrate has shown promise as an adjunctive therapy for various inflammatory conditions, including sepsis. Butyrate is a short-chain fatty acid produced by normal gut microbiota, most notably the *Firmicutes* phylum.^27^ Microbial-derived butyrate limits local inflammation in the colon,^28,29^ while systemic absorption contributes to immune and metabolic homeostasis.^30,31^ In preliminary work, we found that loss of *Firmicutes* organisms from the intestinal microbiota during pediatric sepsis was associated with decreased plasma butyrate levels and correlated with low mitochondrial respiration in blood cells.^32^ We also found that butyrate could prevent and reverse lipopolysaccharide (LPS)-induced mitochondrial dysfunction within in vitro cell lines.^33^ Others have reported that butyrate-producing commensal microorganisms are depleted from the microbiome of septic animals and survival improves after fecal transplant or direct treatment with sodium butyrate.^28,34^

One proposed mechanism of butyrate-induced immunomodulation is augmentation of mitochondrial bioenergetic capacity.^30,35,36^ For example, butyrate supplementation increased mitochondrial respiration in lymphoblastoid cell lines from children with autism^37^ and stimulated mitochondrial biogenesis to increase mitochondrial content in a diabetes mouse model.^30^ However, it is not known if sodium butyrate can directly improve mitochondrial function within vital organ systems during sepsis. We sought to determine if sodium butyrate could prevent or reverse lipopolysaccharide-induced mitochondrial dysfunction in kidney or liver tissue using a mouse model.

## 2 ∣ MATERIALS AND METHODS

### 2.1 ∣ Mice

Nine- to twenty-three-week-old male C57BL/6 wild-type mice purchased from the Jackson or Taconic Laboratories were used for the experiments. Mice were allowed to acclimatize for at least 1 week prior to experiments with unlimited access to food and water. All experiments were approved by the Institutional Animal Care and Use Committees (IACUC) at the Children’s Hospital of Philadelphia and Nemours Children’s Health and complied with the US National Research Council’s Guide for the Care and Use of Laboratory Animals, the US Public Health Service’s Policy on Humane Care and Use of Laboratory Animals, and Guide for the Care and Use of Laboratory Animals. The study design and report adhere to the ARRIVE 2.0 guidelines.

### 2.2 ∣ Experimental procedures

For the first set of experimental conditions using C57BL/6J mice purchased from the Jackson laboratory (RRID:IMSR_JAX:000664), mice were randomly assigned to either intraperitoneal injection of phosphate-buffered saline (PBS) only (control group), lipopolysaccharide (LPS) only, sodium butyrate at doses of 600 or 1200 mg/kg followed 2 h later by LPS, or LPS followed 1 h later by sodium butyrate at doses of 600 or 1200 mg/kg (Figure S1). Sodium butyrate was freshly prepared prior to each experiment and dosing was chosen based on prior data demonstrating optimal survival and neurobehavioral effects at doses between 600 and 1200 mg/kg.^38^ For the second set of experimental conditions using C57BL/6NTac mice purchased from the Taconic Laboratory (RRID:IMSR_TAC:B6), mice were randomly assigned to either intraperitoneal injections of PBS only (control group), LPS only, sodium butyrate 1200 mg/kg followed 2 h later by LPS, LPS followed 1 h later by sodium butyrate 1200 mg/kg, or sodium butyrate 1200 mg/kg only. Because little to no effect was observed with the lower 600 mg/kg dose of sodium butyrate, only the higher butyrate 1200 mg/kg dose was used for the second set of experiments. For all experimental groups, a non-lethal intraperitoneal dose of LPS 5 mg/kg was administered to induce moderate–severe illness but life expectancy of at least 24 h. All mice were euthanized at 24 h after the first intraperitoneal injection by exsanguination via cardiac puncture while under isoflurane anesthesia. Subsequent measurements were conducted with blinding to treatment assignment.

### 2.3 ∣ Tissue homogenization

Following euthanasia, a caudal segment of the left kidney and a segment of the left lateral lobe of the liver were excised and placed in an ice-cold isolation buffer. For the kidney, the capsular outer membrane was carefully pulled back and a triangular ~50 mg specimen that included both cortex and medulla was obtained. Using careful dissection, connective tissue was excised and the remaining tissue was rinsed with isolation buffer to remove residual blood. The remaining tissue was placed in respiration media (MiR05, Oroboros Instruments, Innsbruck, Austria). Using sharp scissors, the tissue was manually minced into tiny pieces and then subjected to a total of 20 strokes in a 2 mL glass Dounce homogenizer. The homogenized kidney sample was transferred to a clean Eppendorf tube and kept on ice until used for respirometry. For liver, visible blood vessels were excised and the remaining tissue was rinsed with isolation buffer to remove residual blood. The remaining tissue was placed in respiration media (MiR05, Oroboros Instruments, Innsbruck, Austria) and minced into tiny pieces using sharp scissors. The tissue was then subjected to a total of 24 strokes in a 2 mL glass Dounce homogenizer. The homogenized sample was centrifuged at 800×*g* for 12 min at 4°C, after which the lipid layer was removed using careful manual pipetting. The final liver homogenate was transferred to a clean Eppendorf tube and kept on ice until used for respirometry. Total cellular protein concentration was determined using the Bradford assay separately for kidney and liver tissue homogenates.

### 2.4 ∣ Mitochondrial respiration

Mitochondrial respiration was measured in freshly prepared kidney (0.25 mg/mL) and liver (1 mg/mL) tissue homogenates as the rate of oxygen consumption per milligram of protein at 37°C using the Oxygraph-2k high-resolution respirometer (Oroboros Instruments, Innsbruck, Austria). Oxygen consumption rate (in pmol O_2_/sec/mg protein) was recorded continuously using DatLab software 7.4 (Oroborus Instruments, Innsbruck, Austria) through a standard substrate-uncoupler-inhibitor-titration protocol, as shown in Figure 1. The sample was added to the Oroboros chamber and oxygen consumption was allowed to stabilize. Complex I substrates glutamate 10 mM, malate 2 mM, and pyruvate 10 mM were added to measure basal CI respiration. Respiration was then stimulated by adding ADP 5 mM to yield maximal oxidative phosphorylation capacity through ETS complex I (OXPHOS_CI_), followed by the addition of succinate 10 mM to measure OXPHOS_CI+II_. Next, the ATP-synthase inhibitor oligomycin was added to measure respiration independent of mitochondrial ATP production in which oxygen consumption was primarily due to leakage of protons across the inner mitochondrial membrane. Maximal OXPHOS-independent oxygen consumption through the ETS was obtained by stepwise titration of carbonyl cyanide m-chlorophenylhydrazone (CCP) to yield ETS_CI+CII_. Addition of the complex I inhibitor, rotenone 0.5 μM, facilitated measurement of ETS_CII_. The ETS was subsequently inhibited by addition of antimycin-A 1 μg/mL (repeated as needed until no further decrease in oxygen consumption was observed), with any residual non-mitochondrial respiration subtracted from other measurements. Finally, the activity of complex IV (CIV) was evaluated by adding ascorbate 2 mM and *N,N,N′,N′*-Tetramethyl-*p*-phenylenediamine dihydrochloride (TMPD) 0.5 mM, an electron donor to complex IV. To account for TMPD autoxidation, sodium azide 40 mM was added, with the difference between the two levels representing CIV-specific respiration.

### 2.5 ∣ Electron transport system complex-specific activity

The specific enzymatic activity of ETS complexes I + III, II + III, and IV was measured on thawed tissue homogenate using established spectrophotometry protocols, with each activity normalized to tissue protein concentration.^39^ Baseline absorbance was measured at 550 nm at room temperature. ETS complex activity was then measured using the rate of change in absorbance during a paired oxidation–reduction reaction initiated by the addition of specific substrates, including NADH for complex I activity, succinate for complex II activity, and reduced cytochrome *c* for CIV activity. Specific enzyme activity (in nmol/min/mg protein) was then calculated using the Beer–Lambert formula with a constant of Є = 18.5 for horse heart cytochrome *c* and cuvette length of 1 cm.

### 2.6 ∣ Mitochondrial content

Mitochondrial content is optimally measured as a percent of cell volume. However, because such measurements can vary between cells and are laborious to calculate, we measured two biochemical markers correlated with mitochondrial content—citrate synthase (CS) activity and mitochondrial DNA relative to nuclear DNA (mtDNA/nDNA). The specific activity of the CS enzyme, part of the tricarboxylic acid cycle, is commonly used to estimate mitochondrial content^40,41^ and was measured using spectrophotometry as the change in absorbance at 412 nm at room temperature after the addition of freshly prepared oxaloacetic acid.^39^ DNA was isolated from kidney and liver tissue homogenate using the DNeasy Blood & Tissue Kit (Qiagen). The mtDNA/nDNA ratio was measured using quantitative real-time PCR performed in triplicate for mtDNA gene ND6 (Mm04225325_g1) and nDNA gene beta-actin (Mm00607939_s1). Data were analyzed using the delta–delta cycle threshold method (ΔΔC_T_) to determine fold-change in the expression of the mitochondrial gene normalized to the nuclear gene in experimental conditions compared to controls.^42^

In addition to total mitochondrial content, expression of individual OXPHOS complex subunits was quantified relative to the mitochondrial content protein voltage-dependent anion channel (VDAC) using Western blotting (Abcam ab110413 and ab154856, Branford, CT). Data were analyzed using the Odyssey XF Imaging System (LI-COR, Lincoln, Nebraska) and presented as the fold-change versus a commercial rodent tissue lysate as a loading control (Abcam ab110341).

### 2.7 ∣ Statistical analysis

Analyses were performed using GraphPad Prism version 9.5.1. Mitochondrial respiration was the primary outcome measure. ETS complex activity, mitochondrial content, and OXPHOS subunit expression were secondary outcomes. Data are presented as means ± standard error (SE) and compared using one-way analysis of variance (ANOVA) with the post hoc Dunnett or Tukey test to correct for multiple comparisons in pairwise comparisons. Statistical significance was defined as a *p*-value ≤.05.

## 3 ∣ RESULTS

A total of 53 mice were initially included, with data available from nine control mice, eight treated with LPS alone (LPS), three pretreated with butyrate 600 mg/kg followed by LPS (B600-LPS), eight pretreated with butyrate 1200 mg/kg followed by LPS (B1200-LPS), three posttreated with butyrate 600 mg/kg after LPS (LPS-B600), seven posttreated with butyrate 1200 mg/kg after LPS (LPS-B1200), and eight treated with butyrate 1200 mg/kg only (B1200). One mouse died prior to 24 h and mitochondrial respiration data from eight mice were noted to be outliers concerning for measurement error; all data from these nine mice were excluded.

### 3.1 ∣ Mitochondrial respiration

In the kidney, mitochondrial respiration was significantly decreased in LPS mice compared to controls for basal CI, OXPHOS_CI+II_, ETS_CI+II_, ETS_CII_, and CIV (Figure 2A–F). Pretreatment with butyrate 600 mg/kg prior to LPS (B600-LPS) did not alter mitochondrial respiration compared to LPS alone, but OXPHOS_CI+II_, ETS_CI+II_, ETS_CII_, and CIV respiration were higher with butyrate 1200 mg/kg pretreatment prior to LPS (B1200-LPS) compared to LPS alone (Figure 2C–F). Posttreatment with butyrate 600 and 1200 mg/kg after LPS (LPS-B600 and LPS-B1200, respectively) did not significantly increase mitochondrial respiration compared to LPS alone, and respiration continued to be lower than controls (Figure 2A,C–F) for all parameters except OXPHOS_CI_ (Figure 2B). Exposure only to butyrate 1200 mg/kg without LPS (B1200) had similar mitochondrial respiration as controls.

Liver mitochondrial respiration was also lower in the liver with LPS compared to controls, although this reduction was not as prominent in the kidney and was not statistically significant across respiratory parameters (Figure 3A–F). Pretreatment with butyrate 1200 mg/kg prior to LPS (B1200-LPS) in the liver demonstrated a similar pattern of increased mitochondrial respiration as in the kidney that were most prominent in OXPHOS_CI+II_, ETS_CI+CII_, and ETS_CII_ (Figure 3C–E), but the differences were not statistically significant. Posttreatment with butyrate 1200 mg/kg after LPS (LPS-B1200) did not improve mitochondrial respiration compared to the LPS group alone.

ETS respiration trended lower in the C57BL/6NTac mice within the control group compared to C57BL/6J mice in both kidney and liver. However, there were no significant differences in ETS respiration between mouse strains across the LPS, B1200-LPS, or LPS-B1200 groups in either the kidney (Figure S2) or the liver (Figure S3).

### 3.2 ∣ Electron transport system complex-specific activity

There were no significant differences in mitochondrial ETS complexes I + II, II + III, or IV within the kidney (Figure 4A) or liver (Figure 4B) for the LPS-treated mice compared to controls. There were also no changes in these specific activities with either pre or posttreatment with butyrate 1200 mg/kg within either kidney or liver (Figure 4A,B).

### 3.3 ∣ Mitochondrial content

CS activity did not differ across groups within either kidney or liver (Figure 5A,B). The mtDNA/nDNA ratio followed a similar pattern as mitochondrial respiration in both kidney and liver with an observed decrease after administration of LPS that was not present with butyrate 1200 mg/kg pretreatment (B1200-LPS) but persisted despite posttreatment with butyrate 1200 mg/kg (LPS-B1200). However, these changes did not reach statistical significance in either kidney or liver (Figure 5C,D).

Representative Western blots for mitochondrial OXPHOS protein subunit expression are shown in Figure 6A,B. In kidney, ETS CI and CII expression were decreased after LPS compared to controls (Figure 6C,D) with an increase in kidney CII with LPS plus butyrate 1200 mg/kg posttreatment (LPS-B1200, Figure 6D). These changes (along with trends in kidney CIII [Figure 6E]) mirrored the pattern of change in kidney mitochondrial respiration (Figure 2B–E), but differed from the absence of change in ETS complex I + III and II + III activity across groups (Figure 4A). There were no differences in kidney CIV and CV expression across groups (Figure 6F,G). There were no differences in liver OXPHOS complex expression across groups (Figure 6H–L).

## 4 ∣ DISCUSSION

In a mouse model of endotoxemic sepsis, we found that pretreatment with high-dose sodium butyrate mitigated the LPS-induced decrease in mitochondrial respiration in the kidney. Posttreatment with sodium butyrate, however, did not significantly reverse the LPS-induced reduction in mitochondrial respiration within the kidney. In the liver, we observed a similar pattern in which pretreatment with high-dose sodium butyrate also mitigated the decrease in mitochondrial respiration induced by LPS, but these differences were not statistically significant, in part because LPS inhibition was less pronounced in the liver compared to the kidney. Posttreatment with sodium butyrate did not reverse the LPS-induced reduction in mitochondrial respiration within the liver. In both the kidney and liver, there was no effect of either LPS or sodium butyrate on mitochondrial ETS complex enzymatic or CS activity. While changes in mtDNA/nDNA also did not reach statistical significance, a notable trend for butyrate pretreatment to blunt a small LPS-induced reduction of this measure of mitochondrial content in both the kidney and liver warrants further investigation, especially since OXPHOS CI and CII subunit expression trended higher with butyrate pretreatment and posttreatment compared to LPS alone in the kidney.

There is increasing evidence that sodium butyrate can augment mitochondrial function through direct use as a mitochondrial fuel, stimulation of intermediary metabolism, attenuation of oxidative stress, upregulation of mitochondrial biogenesis, reduced autophagy, and epigenetic modification through its role as a histone deacetylase inhibitor.^29,30,37,43,44^ In a prior study, we demonstrated that butyrate both prevented and reversed LPS-induced reduction in mitochondrial respiration in Epstein–Barr virus (EBV)-transformed lymphoblast cell lines without effecting CS activity as a marker of mitochondrial content.^33^ Our current data extend these observations to an in vivo protective benefit of pretreatment with high-dose sodium butyrate on mitochondrial respiration within kidney and, possibly, liver tissue. The absence of an effect of butyrate on ETS complex activity in our murine LPS model suggests an indirect mitochondrial benefit rather than direct effect on ETS activity. In kidney, OXPHOS CI and CII subunit expression mirrored the observed changes in respiration with LPS and butyrate pre and posttreatment. These findings suggest that LPS and butyrate are more likely to affect respiration through alterations in mitochondrial complex availability rather than through the specific activity of each ETS complex. However, we are not able draw firm conclusions about the impact of sodium butyrate on mitochondrial content in this model, as we observed no changes in CS activity and only numerical improvement in mtDNA/nDNA ratio with butyrate pretreatment without statistical significance. Moreover, the observed changes in OXPHOS CI and CII subunit expression did not provide clear statistical significance that butyrate improved complex expression. Therefore, whether butyrate preserves mitochondrial content overall (or specific ETS complex expression) through increased biogenesis or reduced mitophagy in this model requires further testing.

While additional work is needed to identify the mechanisms through which sodium butyrate protects mitochondrial function, our study enhances the rationale for butyrate as a therapeutic strategy to combat the deleterious metabolic effects of sterile and infectious inflammation that are associated with the depletion of butyrate-producing organisms from the intestinal microbiome and a reduction in plasma and stool butyrate levels. For example, oral butyrate supplementation, and to a lesser extent colonic butyrate enemas, reduced the severity and increased remission rates for patients with inflammatory bowel disease in clinical trials.^45^ In sepsis, exogenous treatment with butyrate or restoration of a healthy intestinal microbiome that includes butyrate-producing organisms has been shown to restore immunometabolic homeostasis and reduce morbidity and mortality in animal models.^28,34,46^ Although most preclinical and human studies have focused on long-term daily butyrate supplementation over weeks, we demonstrate that even short-term parenteral administration within hours of an acute inflammatory insult can have protective metabolic effects. Our results align with a reduction in mortality, circulating pro-inflammatory cytokines, and intestinal barrier disruption in a rat cecal ligation and puncture model after intravenous treatment with butyrate at 1 and 8 h after the operative insult.^28^

It is reasonable to speculate that the ability of sodium butyrate to limit systemic inflammation is, at least in part, attributable to its protective effects on the mitochondria. We have shown that butyrate can mitigate the LPS-mediated reduction in mitochondrial respiration in the kidney, while others have shown that oxidative stress driven through the mitochondria is attenuated in the heart with butyrate prior to LPS. Mitochondrial dysfunction, as indicated by low respiration and increased oxidative stress, has been linked to increased and persistent systemic inflammation in sepsis and other critical illnesses.^47,48^ Moreover, recovery of mitochondrial function through biogenesis has been associated with reduced inflammation and clinical recovery.^22,49,50^ As such, there is a need to directly evaluate the causal pathways through which butyrate may enhance immunometabolic function and limit the dysregulated inflammatory host response in sepsis.

There are several limitations to our study. First, we used two C57BL/6 mice strains of varying ages. Although this approach added heterogeneity within the experimental model, it is possible that the effects of butyrate varied across different genetic backgrounds. In particular, the C57BL/6J mouse strain, while commonly used, includes a mutation in nicotinamide nucleotide transhydrogenase (NNT) that results in mitochondrial redox abnormalities. Although no significant differences were observed between strains, LPS did appear to impair mitochondrial respiration to a greater extent in the C57BL/6J than in the C57BL/6N strain. Second, we used a relatively low, typically non-lethal dose of LPS to ensure mice would survive for 24 h to facilitate a common timepoint for tissue harvesting. It is possible that the effects of butyrate were reduced by the lower illness severity. Third, the dose of butyrate was selected based on a prior study demonstrating optimal survival and neurobehavioral effects at doses between 600 and 1200 mg/kg in a model of Huntington’s disease with only a single dose administered.^38^ Other studies have used a broader range of doses given via oral, IP, or intravenous routes without a clear consensus of optimal dosing. Fourth, LPS has been shown to induce more severe mitochondrial dysfunction in kidney than liver and the mitochondrial content in liver is lower than in kidney cells, which may have contributed to our limited findings in the liver for respiration and OXPHOS protein subunit expression. Fifth, because CS activity and mtDNA/nDNA cannot differentiate between intact mitochondria and mitochondrial fragments, more direct measures are needed to clarify changes in functional mitochondrial mass with butyrate. Finally, we focused on mitochondrial respiration and content without other measures of mitochondrial quality control, dynamics, and function or direct measures of organ function and inflammation that should be incorporated into future efforts to better understand the mechanisms through which butyrate may mitigate mitochondrial, inflammatory, and organ effects after LPS.

In summary, the results of our study demonstrate that pretreatment with butyrate, a short-chain fatty acid normally produced through the fermentation of carbohydrates by commensal organisms within the intestinal microbiome, can mitigate the LPS-induced decrease in mitochondrial respiration in the kidney and, possibly, the liver. Mitochondrial protection was not attributable to changes in ETS complex activity but may reflect maintenance of ETS subunit expression. In combination with prior studies, our findings support further investigation of the therapeutic potential of sodium butyrate to restore metabolic homeostasis and limit systemic inflammation during sepsis.

## Supplementary Material

Figure S2

Figure S1

Figure S3

Additional supporting information can be found online in the Supporting Information section at the end of this article.

## Figures and Tables

**FIGURE 1:**
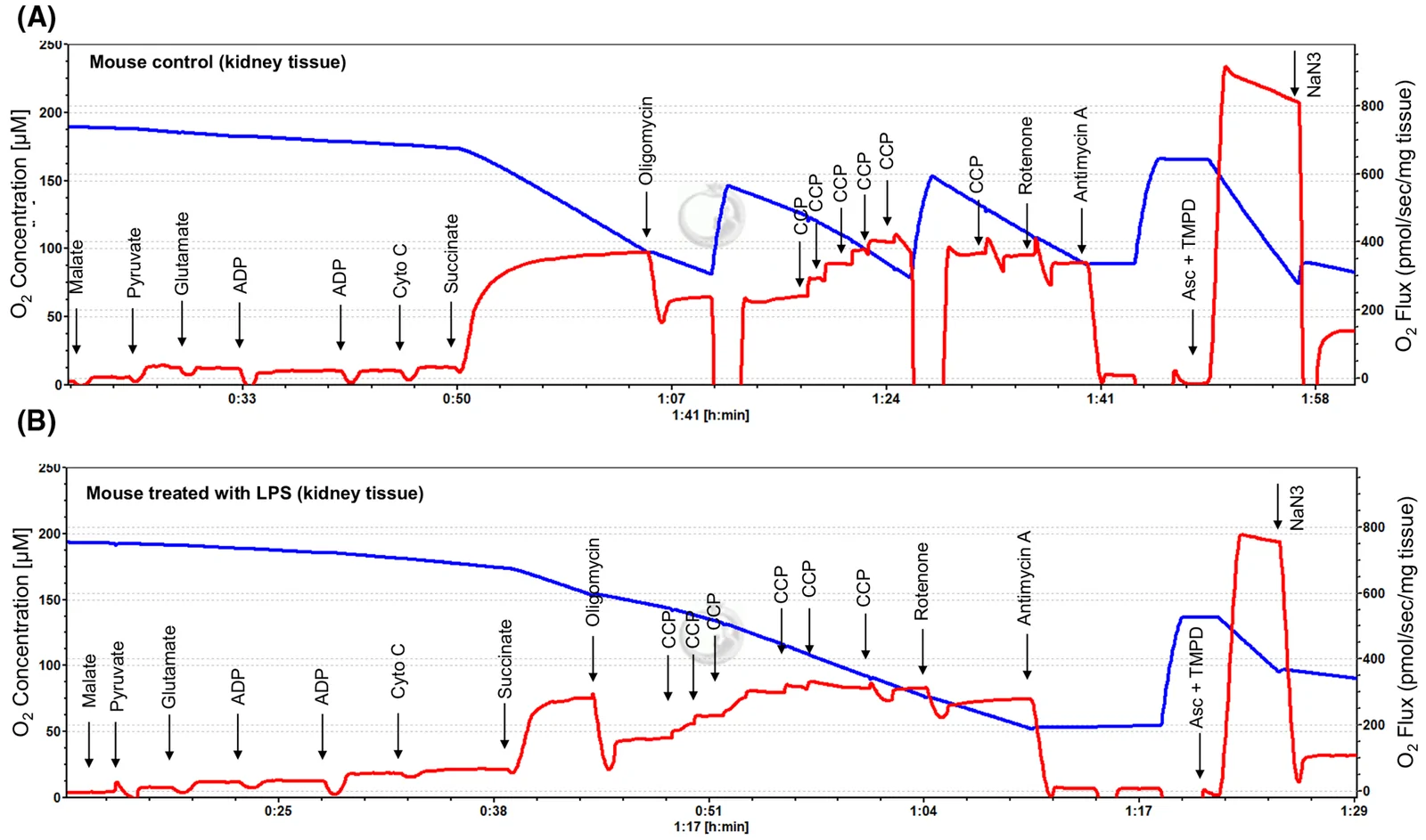
Substrate-inhibitor-uncoupler-titration protocol used to measure mitochondrial respiration. Representation of the substrate-inhibitor-uncoupler-titration protocol used to measure mitochondrial respiration in kidney tissue homogenates from control mice (A) and after LPS (B). The same protocol was used in other experimental groups and in liver tissue homogenates. The blue line indicates oxygen concentration and the red line indicates the rate of oxygen consumption (O_2_ flux) in pmol/sec/mg tissue.

**FIGURE 2:**
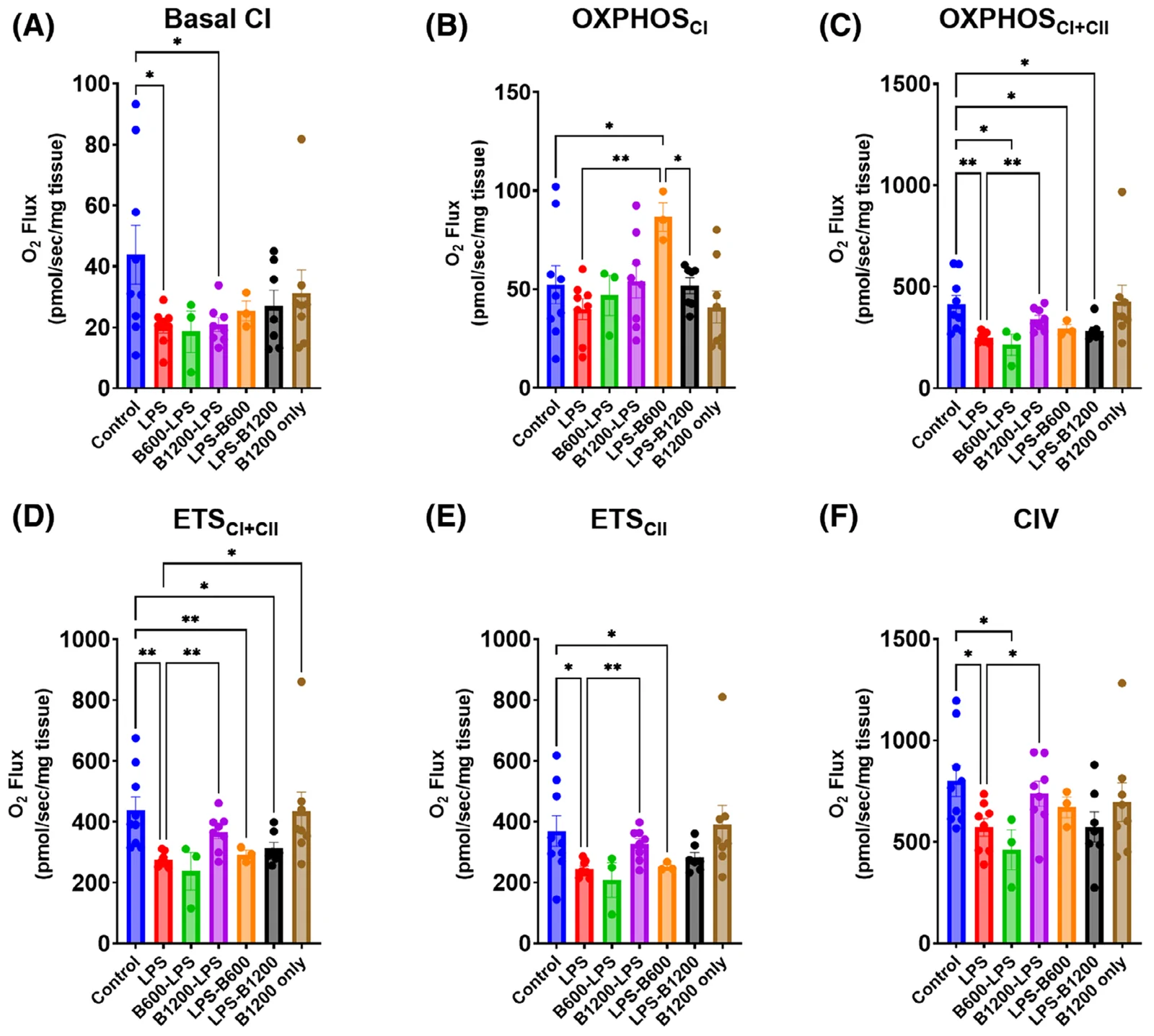
Kidney mitochondrial respiration. Mitochondrial respiration is measured within kidney tissue homogenates using a high-resolution respirometer. Data are presented as oxygen flux in pmol O_2_/sec/mg tissue, which is directly proportional to the oxygen consumption rate. Groups are indicated as control (phosphate-buffered saline only), LPS (lipopolysaccharide) only, B600-LPS (sodium butyrate 600 mg/kg followed 2 h later by LPS), B1200-LPS (sodium butyrate 1200 mg/kg followed 2 h later by LPS), LPS-B600 (LPS followed 1 h later by sodium butyrate 600 mg/kg), LPS-B1200 (LPS followed 1 h later by sodium butyrate 1200 mg/kg), or B1200 (sodium butyrate 1200 mg/kg) only. Graphs A–F indicate distinct parameters of mitochondrial respiration. Data are presented as mean ± SE with individual observations represented by the overlaying circles. **p < .01 and *p < .05 after correction for multiple comparisons using the post hoc Dunnett test.

**FIGURE 3:**
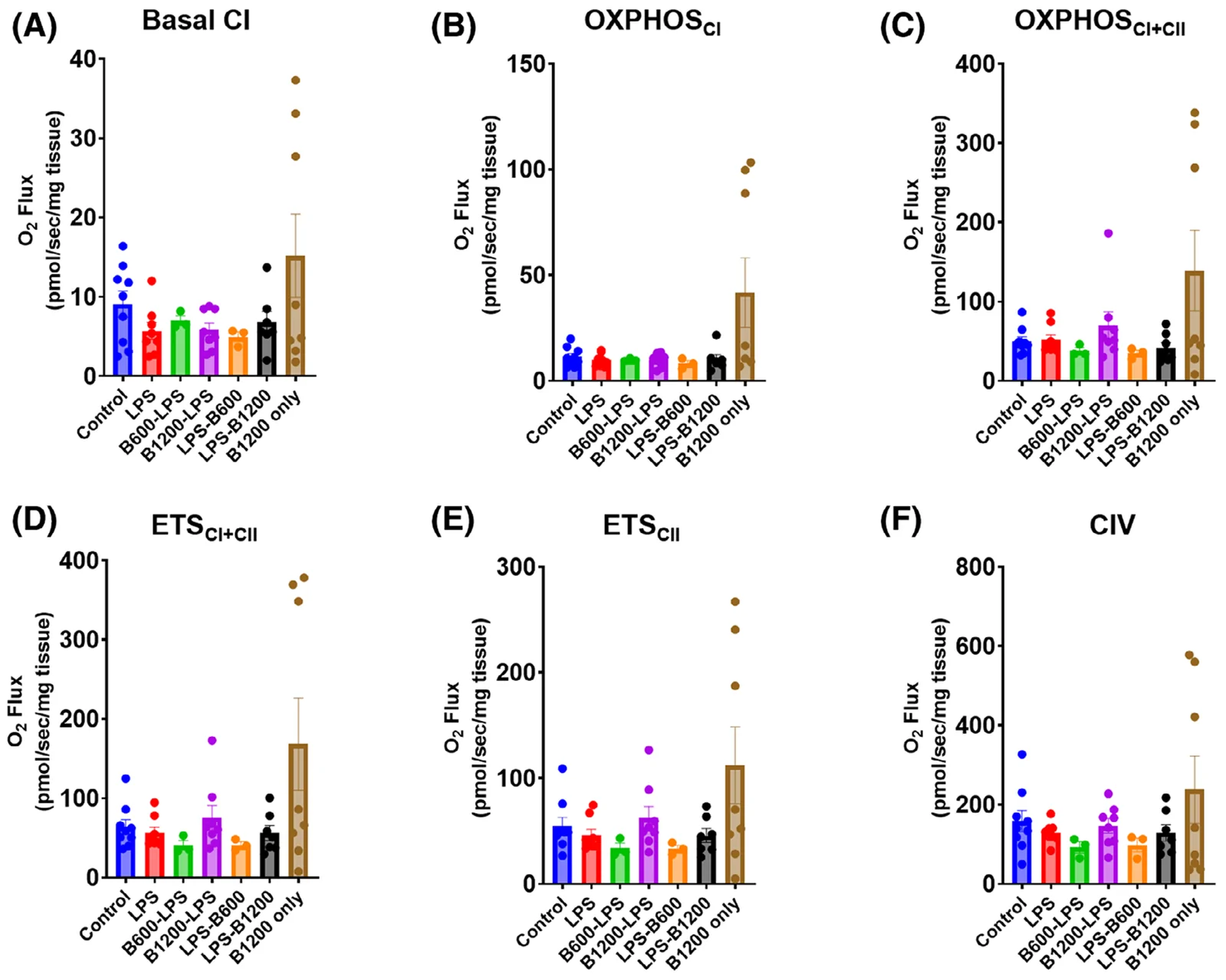
Liver mitochondrial respiration. Mitochondrial respiration is measured within liver tissue homogenates using a high-resolution respirometer. Data are presented as oxygen flux in pmol O_2_/sec/mg tissue, which is directly proportional to the oxygen consumption rate. Groups are indicated as control (phosphate-buffered saline only), LPS (lipopolysaccharide) only, B600-LPS (sodium butyrate 600 mg/kg followed 2 h later by LPS), B1200-LPS (sodium butyrate 1200 mg/kg followed 2 h later by LPS), LPS-B600 (LPS followed 1 h later by sodium butyrate 600 mg/kg), LPS-B1200 (LPS followed 1 h later by sodium butyrate 1200 mg/kg), or B1200 (sodium butyrate 1200 mg/kg) only. Graphs A–F indicate distinct parameters of mitochondrial respiration. Data are presented as mean ± SE with individual observations represented by the overlaying circles. None of the comparisons were significantly different after correction for multiple comparisons using the post hoc Dunnett test.

**FIGURE 4:**
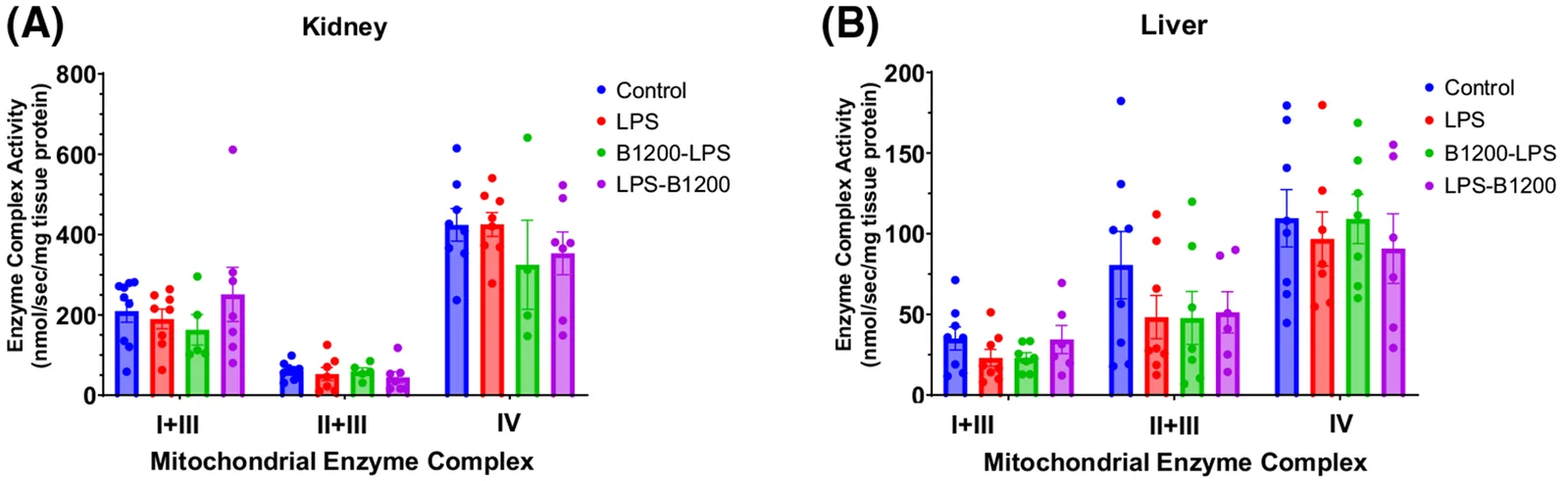
Mitochondrial electron transport system enzyme complex activity. Mitochondrial electron transport system complex activity was measured in (A) kidney and (B) liver tissue homogenates in control (phosphate-buffered saline only), LPS (lipopolysaccharide) only, B1200-LPS (sodium butyrate 1200 mg/kg followed 2 h later by LPS), and LPS-B1200 (LPS followed 1 h later by sodium butyrate 1200 mg/kg). Data are presented as mean ± SE with individual observations represented by the overlaying circles. Mitochondrial electron transport complex I + II, II + III, and IV were not significantly different across groups after correction for multiple comparisons using the post hoc Dunnett test.

**FIGURE 5:**
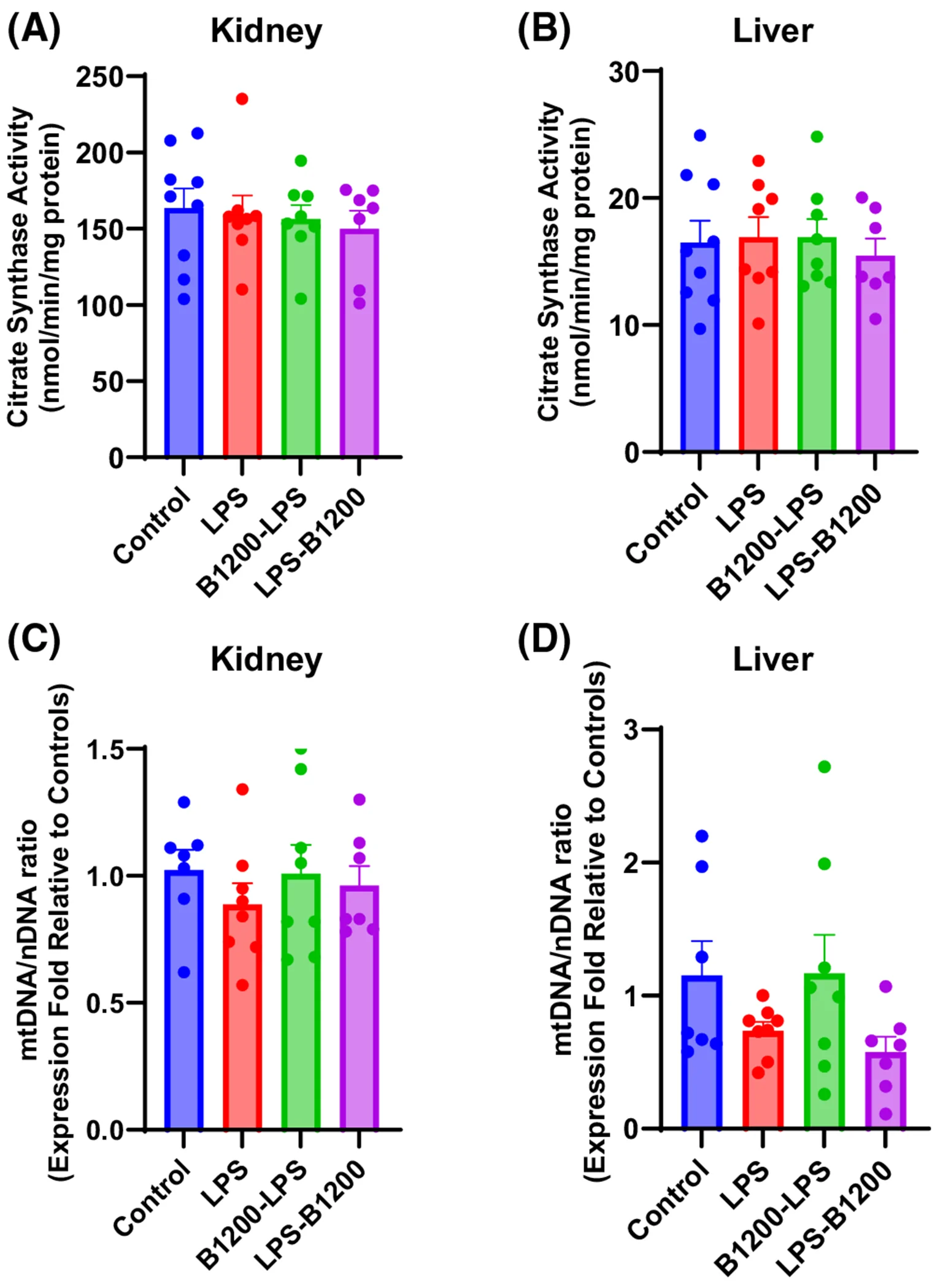
Mitochondrial content. (A, B) Citrate synthase activity as nmol/min/mg cellular protein and (C, D) mtDNA/nDNA presented as expression fold change relative to controls. Data are presented as mean ± SE with individual observations represented by the overlaying circles. None of the comparisons were significantly different after correction for multiple comparisons using the post hoc Dunnett test.

**FIGURE 6:**
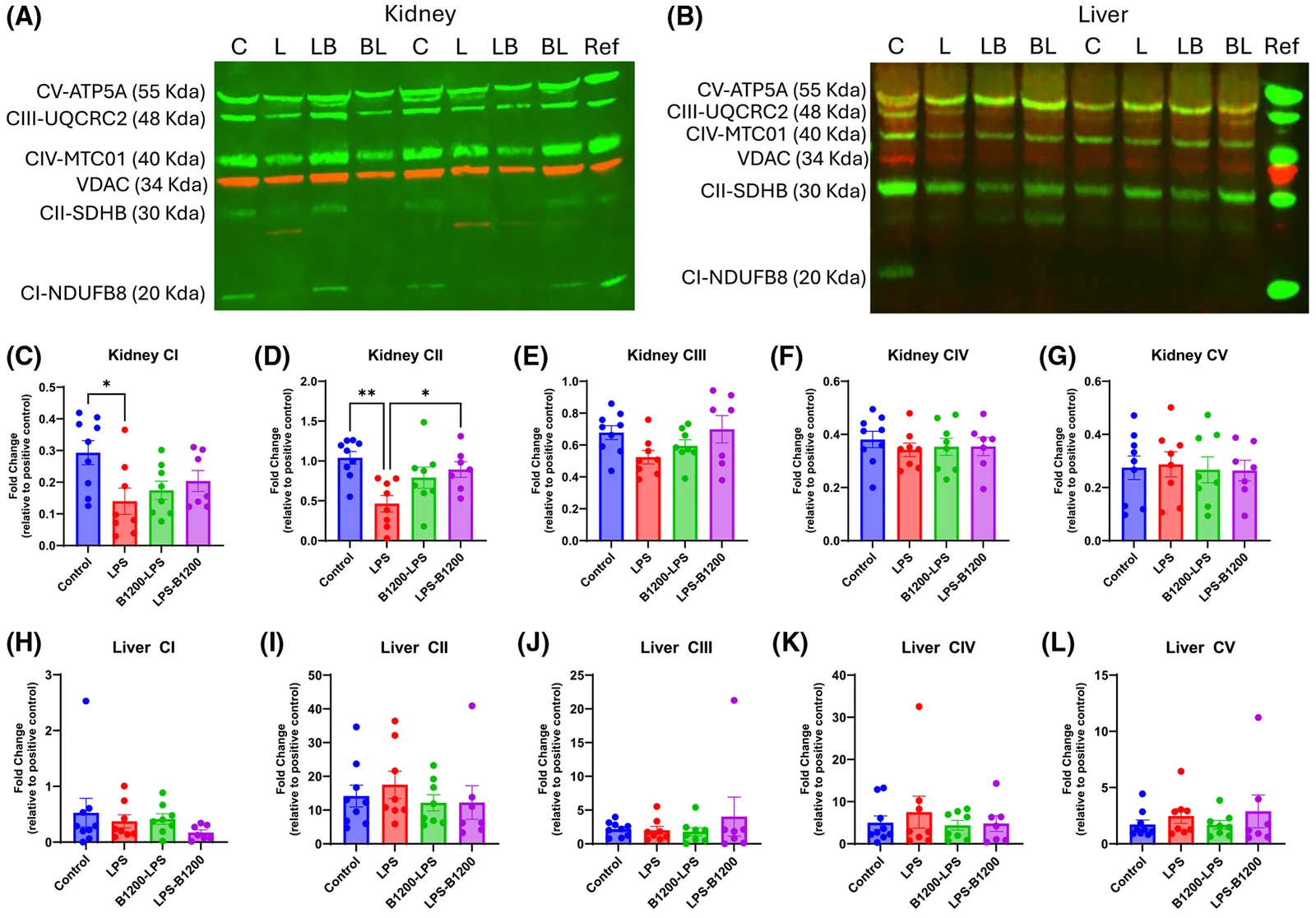
OXPHOS subunit expression. Representative Western blot of mouse kidney (A) and liver (B) homogenates from control (C), lipopolysaccharide (L or LPS) only, LPS followed 1 h later by sodium butyrate 1200 mg/kg (LB or LPS-B1200), sodium butyrate 1200 mg/kg followed 2 h later by LPS (BL or B1200-LPS), and a rodent positive control tissue lysate as a loading control. Graphs C–G show the quantification of protein subunits from mitochondrial complex I (NDUFB8), complex II (SDHB), complex III (UCQCRC2), complex IV (MTCO1), and complex V (ATP5A) in kidney homogenate. Graphs H–L show the mean quantification of mitochondrial complex subunits from liver homogenate. Data are presented as the mean ± SE fold-change relative to the reference loading control with individual observations represented by the overlaying circles. Kidney CI and CII content were decreased after LPS compared to controls with an increase in kidney CII for LPS followed 1 h later by sodium butyrate 1200 mg/kg (LB or LPS-B1200). No other changes in kidney or liver OXPHOS subunit expression were noted. *p < .05, **p < .01 after correction for multiple comparisons.

## Data Availability

The data that support the findings of this study are available on request from the corresponding author. The data are not publicly available due to privacy or ethical restrictions.

## Abbreviations:

ANOVA: analysis of variance
B1200: butyrate 1200 mg/kg
B600: butyrate 600 mg/kg
CI: complex I
CII: complex II
CIII: complex III
CIV: complex IV
CS: citrate synthase
EBV: Epstein-Barr virus
ETS: electron transport system
IACUC: Institutional Animal Care and Use Committees
IP: intraperitoneal
LB: LPS followed 1 hour later by sodium butyrate 1200 mg/kg
LPS: lipopolysaccharide
mtDNA: mitochondrial deoxyribonucleic acid
NADH: nicotinamide adenine dinucleotide + hydrogen
nDNA: nuclear deoxyribonucleic acid
NNT: nucleotide transhydrogenase
OXPHOS: oxidative phosphorylation
PBS: phosphate-buffered saline
SE: standard error
TMPD: *N,N,NȲ,NȲ*-Tetramethyl-p-phenylenediamine dihydrochloride
VDAC: voltage-dependent anion channel
