# Supplementary material for: Effect of sodium butyrate on kidney and liver mitochondrial dysfunction in a lipopolysaccharide mouse model
Source: FASEB J. Author manuscript; Available in PMC 2026 Nov 9. (PMC13746182; doi:10.1096/fj.202401379RR)
Supplement: Supplementary file 1 [file NIHMS2217385-supplement-Figure_S1.pdf]

Figure S1

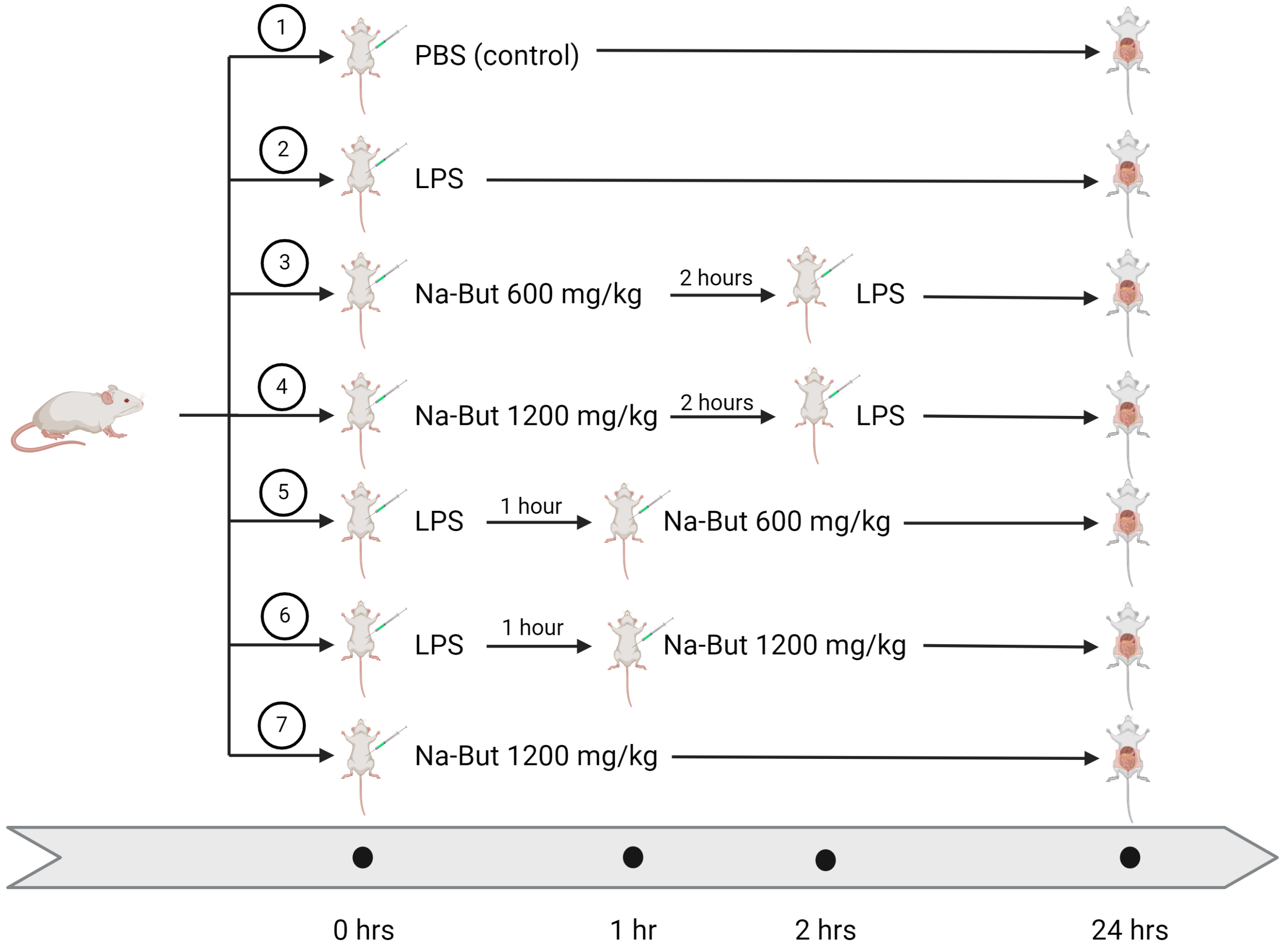


**Supplemental Figure 1: Study Procedures**

Mice were randomly assigned to one of seven groups: (1) phosphate-buffered saline (PBS) only (control group), (2) lipopolysaccharide (LPS) only, (3) sodium butyrate (Na-But) 600 mg/kg followed 2 hours later by LPS, (4) Na-But 1,200 mg/kg followed 2 hours later by LPS, (5) LPS followed 1 hour later by Na-But 600 mg/kg, (6) LPS followed 1 hour later by Na-But 1,200 mg/kg, or (7) Na-But 1,200 mg/kg only. All injections were intraperitoneal (IP). The first IP injection was deemed 0 hours. All mice were euthanized at 24 hours after first IP injection, and kidney and liver tissue were immediately collected for analysis.
