# Supplementary material for: Effect of sodium butyrate on kidney and liver mitochondrial dysfunction in a lipopolysaccharide mouse model
Source: FASEB J. Author manuscript; Available in PMC 2026 Nov 9. (PMC13746182; doi:10.1096/fj.202401379RR)
Supplement: Supplementary file 3 [file NIHMS2217385-supplement-Figure_S3.pdf]

Figure S3

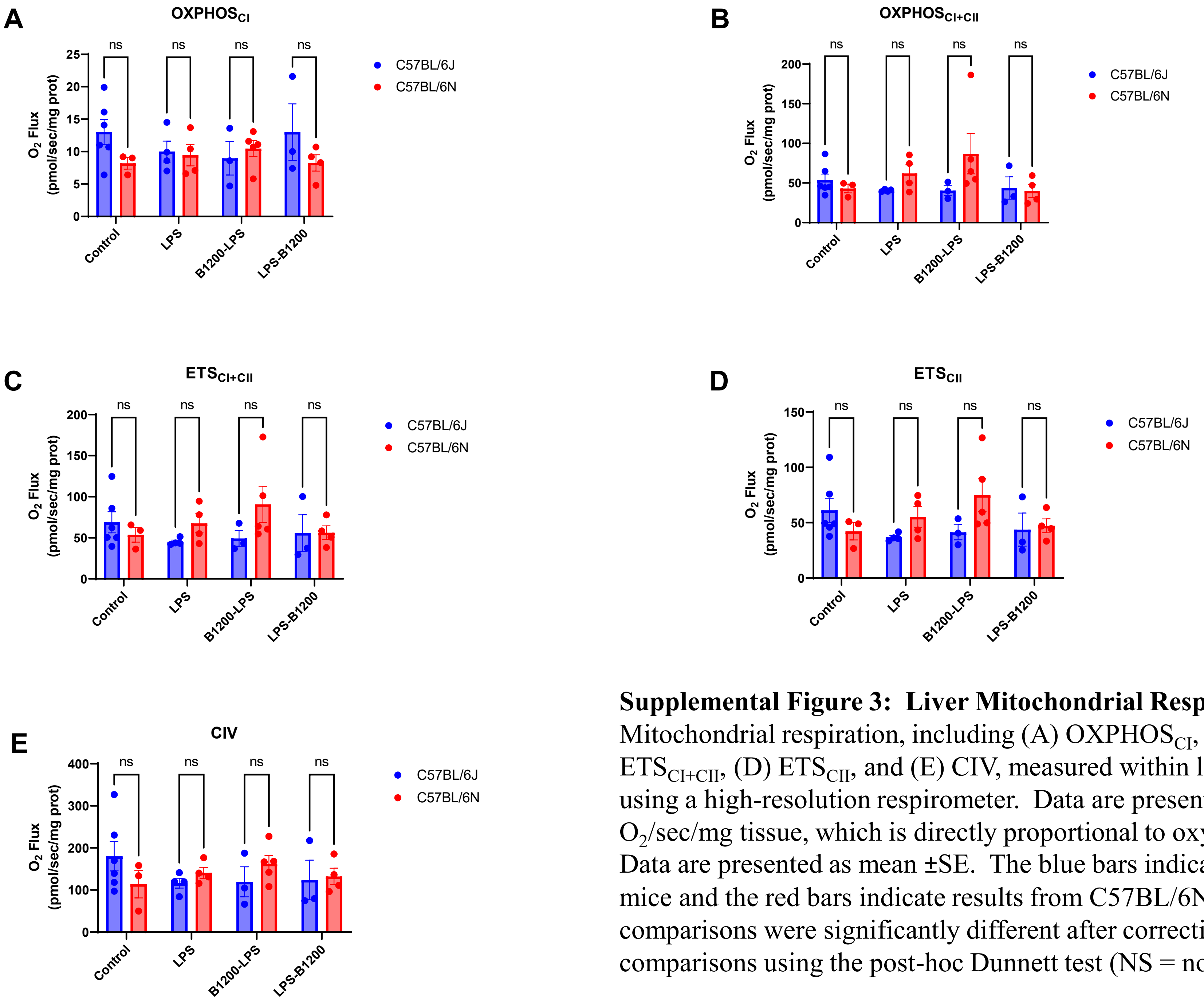

Supplemental Figure 3: Liver Mitochondrial Respiration by Mouse Strain Mitochondrial respiration, including (A) $OXPHOS_{CI}$, (B) $OXPHOS_{CI+CII}$, (C) ETS_{CI+CII}, (D) ETS_{CII}, and (E) CIV, measured within liver tissue homogenates using a high-resolution respirometer. Data are presented as oxygen flux in pmol O_2/sec/mg tissue, which is directly proportional to oxygen consumption rate. Data are presented as mean ±SE. The blue bars indicate results from C57BL/6J mice and the red bars indicate results from C57BL/6N mice. None of the comparisons were significantly different after correction for multiple comparisons using the post-hoc Dunnett test (NS = not significant).
